# Supplementary material for: Survival Benefit of Hepatic Arterial Infusion Chemotherapy over Sorafenib in the Treatment of Locally Progressed Hepatocellular Carcinoma
Source: Cancers (Basel). 2021 Feb 5;13(4):646. doi: 10.3390/cancers13040646 (PMC7915251; doi:10.3390/cancers13040646)
Supplement: Supplementary file 1 [file cancers-13-00646-s001.pdf]

Survival Benefit of Hepatic Arterial Infusion Chemotherapy Over Sorafenib in the Treatment of Locally Progressed Hepatocellular Carcinoma

Authors: Hideki Iwamoto, Takashi Niizeki, Hiroaki Nagamatsu, Kazuomi Ueshima, Takako Nomura, Teiji Kuzuya, Kazuhiro Kasai, Yohei Kooka, Atsushi Hiraoka, Rie Sugimoto, Takehiro Yonezawa, Akio Ishihara, Akihiro Deguchi, Hirotaka Arai, Shigeo Shimose, Tomotake Shirono, Masahito Nakano, Shusuke Okamura, Yu Noda, Naoki Kamachi, Miwa Sakai, Hiroyuki Suzuki, Hajime Aino, Norito Matsukuma, Satoru Matsugaki, Kei Ogata, Yoichi Yano, Takato Ueno, Masahiko Kajiwara, Satoshi Itano, Kunitaka Fukuizumi, Hiroshi Kawano, Kazunori Noguchi, Masatoshi Tanaka, Taizo Yamaguchi, Ryoko Kuromatsu, Atsushi Kawaguchi, Hironori Koga, Takuji Torimura

Supplementary materials

1. Supplementary Table 1
2. Supplementary Table 2
3. Supplementary Table 3
4. Supplementary Table 4
5. Supplementary Table 5
6. Supplementary Table 6
7. Supplementary Table 7
8. Supplementary Table 8
9. Supplementary Table 9
10. Supplementary Table 10
11. Supplementary Table 11
12. Supplementary Figure 1
13. Supplementary Figure 2

14. Supplementary Figure 3

1 Supplementary Table 1. Factors which associate with poor prognosis in the whole data before propensity score matching analysis

Factors	Unit	Odds ratio	95% Confidence interval	p
Group (Sorafenib)	N/A	1.49	1.30–1.70	<0.001
Presence of severe MVI	N/A	1.57	1.37–1.80	<0.001
Presence of EHS	N/A	1.39	1.23–1.58	<0.001
Child-Pugh Class B	N/A	1.79	1.57–2.05	<0.001

Abbreviations: MVI; macrovascular invasion, EHS; extrahepatic spread

2 Supplementary Table 2.

Patient and tumor characteristics of cohort-1

	Cohort-1 n=408		
Patient characteristics	New FP n=73	Sorafenib n=335	*p*-Value
Age (years)	69.38 ± 10.83	72.79 ± 8.29	0.003
Sex<br>Male / Female	59 / 14	261 / 74	0.696
HCV	31 / 42	223 / 112	<0.001
HBV	16 / 57	41 / 294	0.048
Child-Pugh class<br>A / B / C	52 / 20 / 1	288 / 45 / 2	0.009
Tumor characteristics			
Tumor size (mm)	110.93± 52.76	92.47± 54.14	0.008
MVI	0 / 73	0 / 335	
EHS	0 / 73	0 / 335	
AFP (ng/ml)	617.03± 359.82	610.72± 340.07	0.887
DCP (mAU/ml)	618.70± 337.23	592.51± 335.61	0.549

Abbreviation MVI: macrovascular invasion, EHS: extrahepatic spread, AFP: alpha-phetoprotein, DCP: des-gamma carboxyprothrombin

3 Supplementary Table 3.

Factors which associate with poor prognosis in cohort-1

Factors	Unit	hazard ratio	95% Confidence interval	p
Group (Sorafenib)	N/A	1.44	1.01-2.04	0.043
Maximum tumor diameter (mean)	5	1.01	1.00-1.02	0.035

4 Supplementary Table 4

Patient and tumor characteristics of cohort-2

	Cohort-2 n=591		
Patient characteristics	New FP n=442	Sorafenib n=149	*p*-Value
Age (years)	68.38 ± 10.44	67.78 ± 10.19	0.541
Sex<br>Male / Female	344 / 247	112 / 37	0.578
HCV	218 / 224	68 / 81	0.494
HBV	79 / 363	38 / 111	0.057
Child-Pugh class<br>A / B / C	280 / 148 / 14	119 / 30 / 0	<0.001
Tumor characteristics			
Tumor size (mm)	113.84± 51.91	109.79± 52.17	0.410
MVI	442 / 0	149 / 0	
EHS	0 / 442	0 / 149	
AFP (ng/ml)	630.92± 364.45	604.91± 328.83	0.441
DCP (mAU/ml)	581.72± 348.07	581.55± 368.86	0.996

Abbreviation MVI: macrovascular invasion, EHS: extrahepatic spread, AFP: alpha-phetoprotein, DCP: des-gamma carboxyprothrombin

5 Supplementary Table 5.

Factors which associate with poor prognosis in cohort-2

Factors	Unit	hazard ratio	95% Confidence interval	p
Group (Sorafenib)	N/A	1.97	1.58-2.44	<0.001
Child-Pugh class B	N/A	2.01	1.631-2.43	<0.001
Presence of severe MVI	N/A	1.43	1.18–1.74	<0.001

Abbreviation: MVI macrovascular invasion

6. Supplementary Table 6.

Comparison of median survival time in Cohort-2 (After PSM data)

	New FP (n=148)	Sorafenib (n=148)	p
MST (Months)	15	7.9	<0.001

Abbreviations: PSM; propensity score matching, MST; median survival time

7 Supplementary Table 7.

Patient and tumor characteristics of cohort-3

	Cohort-3 n=369		
Patient characteristics	New FP n=13	Sorafenib n=356	*p*-Value
Age (years)	64.38 ± 16.20	70.04 ± 8.99	0.032
Sex<br>Male / Female	8 / 5	278 / 78	0.287
HCV	8 / 5	201 / 155	0.938
HBV	2 / 11	61 / 295	1.000
Child-Pugh class<br>A / B / C	8 / 5 / 0	299 / 57 / 0	0.001
Tumor characteristics			
Tumor size (mm)	112.00± 60.71	63.49± 63.21	0.007
MVI	0 / 13	0 / 369	
EHS	13 / 0	369 / 0	
AFP (ng/ml)	621.85± 341.93	614.43± 364.92	0.943
DCP (mAU/ml)	792.15± 313.98	601.24± 349.72	0.053

Abbreviation: MVI: macrovascular invasion, EHS: extrahepatic spread, AFP: alpha-phetoprotein, DCP: des-gamma carboxyprothrombin

8 Supplementary Table 8.

Factors which associate with prognosis in cohort-3

Factors	Unit	hazard ratio	95% Confidence interval	p
Maximum tumor diameter (mean)	5	1.03	1.02-1.04	<0.001
Child-Pugh class B	N/A	1.75	1.27-2.39	0.001
HBV	N/A	0.67	0.47–0.95	0.023

9 Supplementary Table 9

Patient and tumor characteristics of cohort-4

	Cohort-4 n=256		
Patient characteristics	New FP n=116	Sorafenib n=140	*p*-Value
Age (years)	65.74 ± 12.33	66.37 ± 10.91	0.665
Sex<br>Male / Female	94 / 22	120 / 20	0.403
HCV	43 / 73	54 / 86	0.907
HBV	26 / 90	39 / 101	0.394
Child-Pugh class<br>A / B / C	65 / 45 / 6	103 / 37 / 0	0.001
Tumor characteristics			
Tumor size (mm)	99.80± 54.66	108.32± 55.46	0.219
MVI	116 / 0	140 / 0	
EHS	116 / 0	140 / 0	
AFP (ng/ml)	603.39± 350.01	602.36± 365.80	0.982
DCP (mAU/ml)	588.07± 345.94	629.74± 364.81	0.360

Abbreviation: MVI: macrovascular invasion, EHS: extrahepatic spread, AFP: alpha-phetoprotein, DCP: des-gamma carboxyprothrombin

10 Supplementary Table 10.

Factors which associate with poor prognosis in cohort-4

Factors	Unit	hazard ratio	95% Confidence interval	p
Presence of severe MVI	N/A	1.49	1.22-2.12	0.001

Abbreviation: MVI: macrovascular invasion

11. Supplementary Table 11.

Comparison of median survival time in Cohort-4 (After PSM data)

	New FP (n=94)	Sorafenib (n=94)	p
MST (Months)	8	5	0.089

Abbreviations: PSM; propensity score matching, MST; median survival time

Supplementary Figure and Figure legends

12 Supplementary Figure 1.

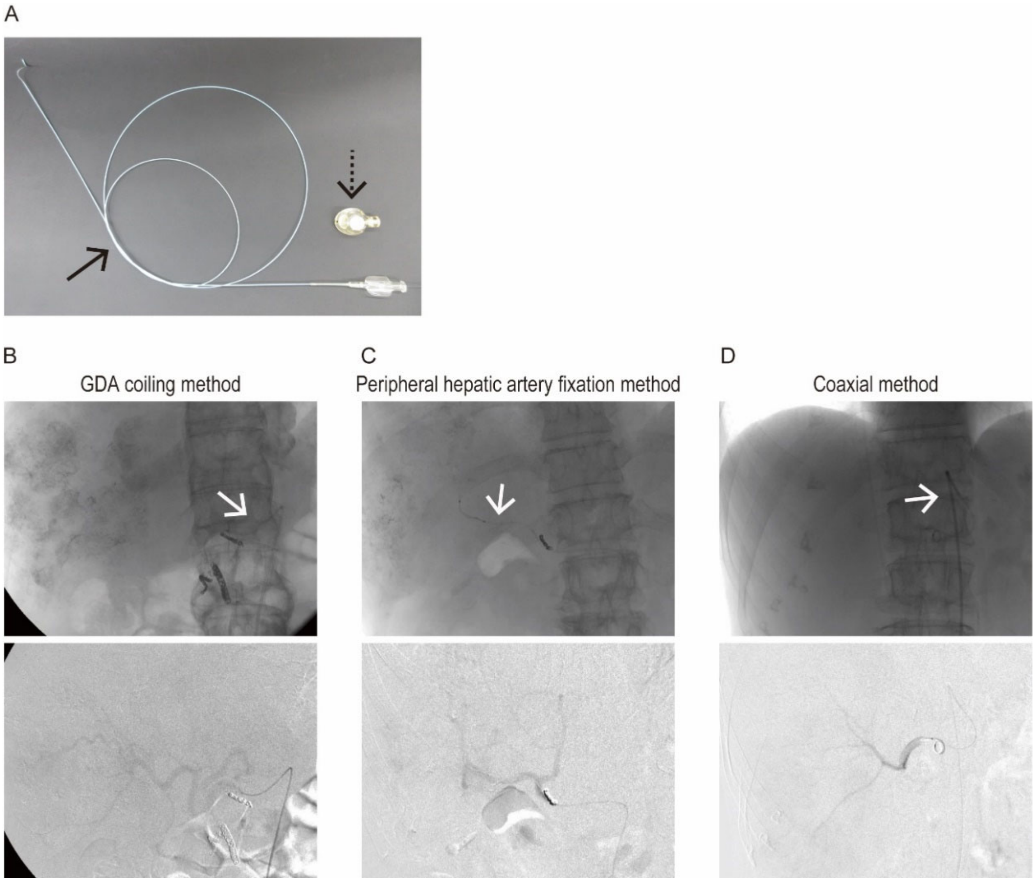

Catheter implantation procedures for HAIC

A. The image of an implanted catheter for HAIC treatment in the study.
   Arrow shows the indwelling catheter for HAIC. Dashed arrow shows the port for HAIC.

B. The various indwelling methods of the implanted catheter in the study. The GDA coiling method. Arrow shows the implanted catheter.

C. The peripheral hepatic artery fixation method. Arrow shows the implanted catheter.

D. The coaxial method. Arrow shows the implanted catheter.

HAIC: hepatic arterial infusion chemotherapy, GDA: gastroduodenal artery

13 Supplementary Figure 2.

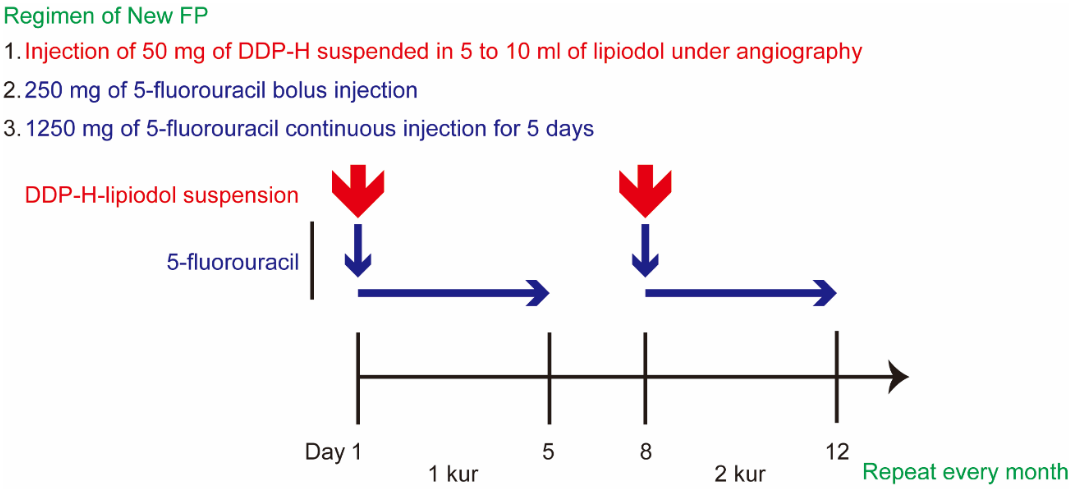

A HAIC regimen in New FP

The fine-powder formulation of CDDP was used in New FP regimen (DDP-H, IA-Call, Nippon Kayaku, Tokyo, Japan). As the inpatient regimen of New FP, 50 mg of fine-powder CDDP was suspended in 5-10 ml of lipiodol of which the amount was decided by tumor volume. At day 1, DDP-H-lipiodol suspension was injected from the implanted catheter under the angiography, followed by 250 mg of 5-FU was injected. Then, 1250 mg of 5-FU was continuously injected using an infusion balloon pump for 5 days (SUREFUSER PUMP, Nipro Pharma Corporation, Osaka Japan). This regimen was applied once a week during the first two or three weeks. As the outpatient regimen of New FP, 20 to 30 mg of DDP-H-lipiodol suspension was injected under the angiography, followed by 1250 mg of 5-FU was injected using an infusion balloon pump for 5 days at every two weeks. The inpatient regimen of New FP was administered by depending on the time course of tumor progression, on demand. These regimens were continued until the appearance of severe adverse events or tumor progression.

HAIC: hepatic arterial infusion chemotherapy, CDDP: cisplatin, 5-FU: 5-fluorouracil

14 Supplementary Figure 3.

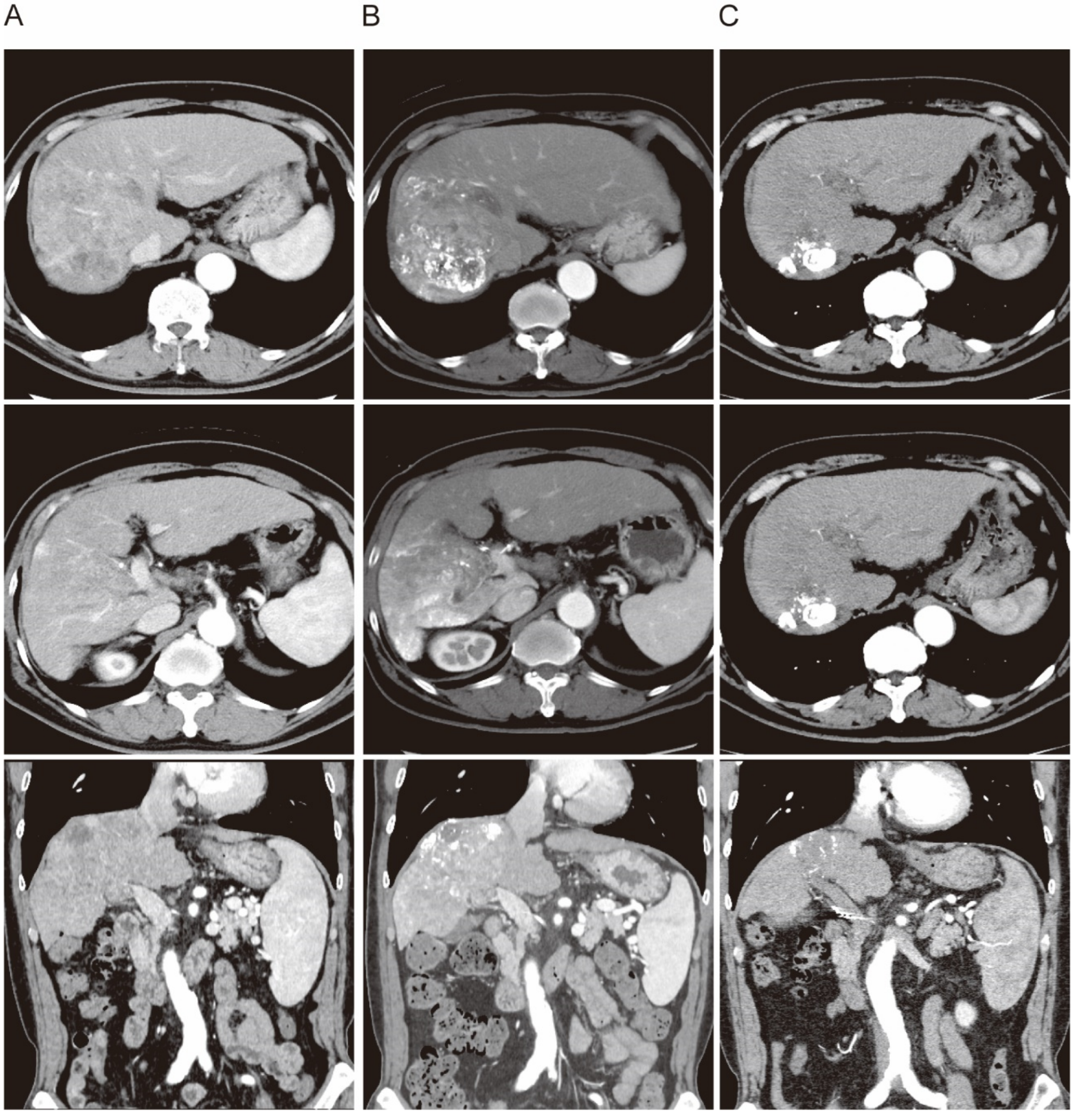

The time course of the representative CT images in New FP therapy

A. The image before New FP therapy. 10 cm of HCC is located on hepatic segment 5/8.

B. The image after one time of New FP therap. The injected DDP-H-lipiodol suspensions are accumulated into tumors.

C. The image after 4 months of New FP therapy. Complete necrosis was achieved after 10 times of New FP therapy for 4 months.
